# Are There Modifiable Risk Factors to Improve AKI?

**DOI:** 10.1155/2017/5605634

**Published:** 2017-07-04

**Authors:** Sasa Nie, Li Tang, Weiguang Zhang, Zhe Feng, Xiangmei Chen

**Affiliations:** Department of Nephrology, Chinese PLA General Hospital, Chinese PLA Institute of Nephrology, State Key Laboratory of Kidney Diseases, National Clinical Research Center for Kidney Diseases, Beijing Key Laboratory of Kidney Diseases, Beijing 100853, China

## Abstract

Acute kidney injury (AKI) is a common critical syndrome, with high morbidity and mortality. Patients with AKI typically have an adverse prognosis, from incident chronic kidney disease (CKD), progression to end-stage renal disease (ESRD), subsequent cardiovascular disease, and ultimately death. However, there is currently no effective therapy for AKI. Early detection of risk factors for AKI may offer a good approach to prevention or early intervention. Traditional risk factors include extreme age, many common comorbid diseases, such as preexisting CKD, some specific exposures, such as sepsis, and exposure to some nephrotoxic agents. Recently, several novel risk factors for AKI, such as hyperuricemia, hypoalbuminemia, obesity, anemia, and hyperglycemia, have been identified. The underlying mechanisms between these nontraditional risk factors and AKI and whether their correction can reduce AKI occurrence remain to be clarified. This review describes the current epidemiology of AKI, summarizes its outcome, outlines the traditional risk profile, and finally highlights some recently identified novel risk factors.

## 1. Introduction

Acute kidney injury (AKI) is a clinical syndrome of various etiologies, characterized by an abrupt decrease in kidney function, the accumulation of metabolic waste in the body, water-electrolyte and acid-base balance disorders, and systemic symptoms. For a long time, AKI had been lacking a uniform definition and standards. The Acute Dialysis Quality Initiative (ADQI) group put forward the concept of AKI and the RIFLE criteria in 2002. Then, the Acute Kidney Injury Network (AKIN) developed the AKIN standard in 2004, and, later, the Kidney Disease: Improving Global Outcomes (KDIGO) group released AKI Clinical Practice guidelines in 2012 [1]. These efforts provide a more unified definition and diagnostic criteria, laying a substantial foundation for practice, research, and public health matters [2]. Thus, the epidemiological characteristics of and risk factors for AKI can be explored and discovered. In this review, we describe the epidemiology and outcomes of AKI and then summarize its novel risk factors to highlight better prevention and interventions for AKI.

## 2. Epidemiology

AKI is a common critical illness with high morbidity and mortality. A systematic review, published in 2013, concerned 312 studies and 49 million patients, primarily in hospital settings [3]. The pooled morbidity and mortality for AKI were 21.6% and 23.9% in adults, respectively, based on the KDIGO AKI definition. However, most of the studies originated from high-income countries; few were from low- and middle-income countries. In 2015, another review added over 300 research results, the combined sample size of which was more than 77 million individuals [4]. This contained some low- and middle-income countries. The pooled morbidity and mortality for AKI were both 21% in hospital admissions. Thus, the prevention and treatment of AKI have become a global public health problem.

The incidence of AKI and those requiring dialysis has increased gradually. Mortality has declined steadily but remains at a high level. Using the Nationwide Inpatient Sample, the incidence of acute renal failure (ARF) in 2002 was 4.7 times that in 1988. Furthermore, those requiring dialysis increased nearly sevenfold between 1988 and 2002, while in-hospital mortality decreased, from 40.4% to 20.3% (*p* < 0.001) [5]. The epidemiology of hospitalized AKI not requiring dialysis in England from 1998 to 2013 showed an increase in morbidity (317 to 3995 pmp), but a decrease in case-fatalities (42.3 to 27.1%; *p* < 0.001) [6]. Regarding the higher morbidity, one explanation is the higher frequency of comorbidities, such as diabetes and chronic kidney disease (CKD), which contribute to the development of AKI. Patients are often exposed to multiple potentially nephrotoxic medications and invasive procedures. Patients with AKI increasingly consist of the elderly. Additionally, nonnephrologists now have an increased awareness of the potential presence of AKI. The reasons for the decline in mortality in AKI patients remain unknown. Possible reasons might be specific interventions or therapies for AKI patients, improvements in patient care, or other reasons [7]. Nevertheless, there is no doubt that AKI patient mortality is still high.

## 3. Outcomes

AKI is associated with an increased risk of incident CKD and end-stage renal disease (ESRD). AKI is also an independent risk factor for death generally. Lo et al. conducted a study in a community-based cohort of patients with normal or near-normal initial kidney function. It showed that dialysis-requiring ARF patients, compared with non-ARF patients, had a 28-fold increased risk in the development of CKD 4-5 and more than a twofold increased risk of death [8]. Wald et al. showed that AKI patients requiring in-hospital dialysis had an incidence rate of chronic dialysis of 2.63 per 100 person-years, whereas those without AKI or dialysis during hospitalization had a rate of 0.91 per 100 person-years. The adjusted hazard ratio was 3.23 (95% confidence interval, CI = 2.70–3.86) [9]. Coca et al. searched the MEDLINE and EMBASE databases between January 1985 and February 2011 to summarize the relationship between CKD, ESRD, death, and AKI. They found that patients with AKI had an 8.8-fold higher risk of developing CKD, a 3.1-fold higher risk of ESRD, and double the risk of premature death compared to those without AKI [10]. The mortality risk increased with the degree of AKI severity [11]. The odds ratio for stage I was 2.2 (95% CI = 2.17–2.30), for stage II, it was 6.1 (95% CI = 5.74–6.44), and for stage III it was 8.6 (95% CI = 8.07–9.15). The severity of AKI predicts progression to CKD, especially for those requiring dialysis and then recovering [12].

The possible mechanisms [13] of CKD progression after AKI include the following.

*(1) Nephron Loss and Remaining Nephrons Hypertrophy*. After loss of nephron mass, the glomeruli will become hypertrophic, which will ultimately lead to tubulointerstitial fibrosis and further nephron loss [14].

*(2) Interstitial Inflammation and Fibrosis.* Interstitial immune cell infiltration can lead to interstitial fibrosis. Tubulointerstitial fibrosis is the predominant pathology both in human CKD and in the remnant kidney [14], and tubulointerstitial fibrosis injury scores are better for the assessment of renal function progression, compared with glomerular injury scores [13].

*(3) Endothelial Injury and Capillary Rarefaction.* Endothelial and vascular injury involved in AKI. Diminished Vascular density is demonstrated in various different injury models [15, 16], because of its worse regenerative capacity, resulting in insufficient blood supply. Hypoxia ensues, ultimately accelerating inflammation and fibrosis. Thus, peritubular capillary loss is hypothesized to play an important role in fibrogenesis and CKD progression after AKI [13].

*(4) Maladaptive Repair (Epithelial Cell Cycle Abnormalities, Epigenetic Changes)*. Injury may follow by a maladaptive repair process; a number of proximal tubule epithelial cells will arrest in the G2/M phase of the cell cycle [17], which is termed as “cell cycle arrest.” Cell cycle arrest leads tubular epithelial cells to epigenetic changes, which will promote the activation and growth of fibroblasts. Epigenetic changes in fibroblasts may ensue, resulting in its tumor-like myofibroblasts transformation [18].

However, we cannot yet conclude that AKI truly leads to CKD [19]. Although all of the studies included in the meta-analysis have adjusted for demographic, physiological, and clinical variables, some unknown residual confounders likely remain unadjusted. The measurements of known confounders might be imprecise, which will affect the judgement of the relationship between AKI and subsequent CKD. The different definitions of AKI and its outcome result in high statistical heterogeneity, which will reduce the reliability of the statistical results. The conclusion that AKI leads to CKD is based mainly on observational studies. Many risk factors for CKD are the same as those of AKI. For CKD patients, we cannot distinguish whether the CKD is caused by AKI or the shared risk factors, because the development of AKI often precedes that of CKD. For patients with AKI on CKD, if the CKD is undetected before AKI, we may mistakenly believe that the CKD is the sequel of AKI, even after the kidney function returns to baseline. Whether AKI is the cause of CKD can be confirmed by randomized, controlled trials, if they show that a reduction of AKI in turn reduces the incidence of CKD.

AKI is also recognized as an important and independent risk factor for many nonrenal outcomes, such as cardiovascular disease. James et al. [20] investigated patients undergoing coronary angiography in a retrospective study of 14,782 adults. Mild AKI was associated with a 1.47-fold increased risk of myocardial infarction (MI), and a 1.48-fold increased risk of congestive heart failure (CHF). Moderate-to-severe AKI was associated with more than a twofold increased risk of CHF. Gammelager et al. [21] explored the influence of AKI on cardiovascular disease in ICU patients. The 3-year cumulative risk of heart failure in patients without AKI, with mild AKI, and with moderate-to-severe AKI was 2.2, 5.0, and 5.0%, respectively. The 3-year cumulative MI risk in patients without AKI, with mild AKI, and with moderate-to-severe AKI was 1.0, 1.8, and 2.3%, respectively. Mild AKI was associated with a 1.33 (95% CI = 1.06–1.66) times increased occurrence of HF, but not for MI. Moderate-to-severe AKI was also associated with a 1.45 (95% CI = 1.14–1.84) times increased risk for heart failure (HF) and a 1.51 (95% CI = 1.05–2.18) times increased risk for MI. However, independent impact of AKI on stroke was unfound.

AKI also has adverse impacts on healthcare costs, adding a large financial burden. A study using data from the National Health Service (NHS) in England estimated that the annual cost on AKI care for inpatients was £1.02 billion, slightly over 1% of the NHS budget [22]. The estimated lifetime cost for AKI patients after hospital discharge in 2010-2011 was £179 million. A large cross-sectional survey of AKI in China showed that all associated hospitalization expenses ($13 billion) represented up to 10% of China's total medical expenses [23].

## 4. Risk Factors

Because there is currently no effective therapy for AKI, factors predicting AKI may be of benefit in optimizing hospital care and preventing AKI in patients. Patients' susceptibility, type and extent of exposure, and/or their interaction determine the risk of AKI. Many studies have contributed to our current understanding of the risk factors for AKI. Age is one “traditional” factor affecting the incidence of AKI. Studies have reported that both elderly and very young patients are particularly inclined to suffer from this disorder [24, 25]. CKD is a major risk factor for AKI [26]. Some nonrenal comorbid diseases, including diabetes mellitus, hypertension, cardiovascular disease, chronic liver disease, and chronic obstructive pulmonary disease, have been implicated as significant predictors of AKI [1]. Many specific exposures can modify the risk of AKI, among which the most important are sepsis, cardiac surgery, and major noncardiac surgery [1]. Exposure to nephrotoxins is associated with AKI [4]. However, these variables are readily intuited and well known to clinicians. Recent research has identified several nontraditional, largely unsuspected, and potentially modifiable risk factors for AKI. Identifying these risk factors could be of substantial benefit.

### 4.1. Hyperuricemia and AKI

Uric acid is the end product of dietary and endogenous purine metabolism. It has been linked historically to AKI secondary to tumor lysis syndrome. However, recent studies have suggested that elevated serum uric acid (SUA) levels may be associated with the risk of AKI in several settings. The main evidence for an association between raised SUA and AKI stems from patients undergoing cardiopulmonary bypass (CPB) surgery. A preliminary study [27] conducted in 58 patients showed that an elevated preoperative uric acid (defined as > 6 mg/dL) conferred a fourfold higher risk for AKI (OR = 3.98, 95% CI = 1.10–14.33, *p* = 0.035). The same investigators confirmed this in a retrospective study of 190 patients after adjusting for major confounding risk factors [28]. They also demonstrated for the first time that SUA and AKI had a J-shaped relationship. In a large observational study of 1,019 patients in a tertiary level university hospital, the odds ratio of developing AKI with an elevated uric acid level of > 6.5 mg/dL was 1.46 (95% CI = 1.04–2.06) [29]. A larger investigation, including 2,185 patients, concluded that preoperative uric acid was significantly related to postoperative AKI (OR = 1.18, 95% CI = 1.10–1.26) and improved the predictive ability for AKI [30]. Whether postoperative SUA can predict AKI has also been assessed. In a prospective, observational study, SUA levels in 100 cardiac surgery patients were determined 24 h postoperatively [31]; compared with the 1st tertile (SUA < 4.53 mg/dL), the 3rd tertile SUA (SUA > 5.77 mg/dL) was associated with an eightfold increased risk of AKI on postoperative day 2 (adjusted OR = 7.94, 95% CI = 1.50–42.08, *p* = 0.015) and a 5-fold increased risk of AKI during the hospital stay (OR = 4.83, 95% CI = 1.21–19.20, *p* = 0.025). These findings suggest that preoperative measurements of SUA levels may help in stratifying risks for AKI in these patients. Moreover, uric acid seems to predict the progression of AKI in patients undergoing cardiac surgery better than neutrophil gelatinase-associated lipocalin (NGAL) [32].

A high SUA has also been reported to be an independent predictor of CI-AKI in some studies [33], but not in others [34]. A systematic review and meta-analysis of 18 relevant studies involving 13,084 participants [35] revealed that the presence of hyperuricemia was associated with an increased risk of CI-AKI development, regardless of whether the effect size was adjusted for (unadjusted OR = 2.08, 95% CI = 1.63–2.64, adjusted OR = 1.68, 95% CI = 1.38–2.04). Another meta-analysis showed that subjects with higher SUA level had a twofold increased risk of developing AKI (pooled OR = 2.03, 95% CI = 1.48–2.78) [36]. The use of allopurinol for preventing the development of CI-AKI has been evaluated in two randomized, controlled trials, lowering SUA with saline hydration, and comparing saline hydration alone versus saline hydration with N-acetyl cysteine. Two clinical trials have suggested that urate-lowering therapy has beneficial effects in reducing the incidence of CI-AKI [37, 38].

Data from a prospective cohort study of medical intensive care unit (MICU) patients with sepsis showed that the probability of having hyperuricemia along with AKI was 68.5% and without AKI was 31.5%. The study showed that elevated uric acid levels on arrival at the ICU were associated with an increased risk of AKI [39].

Recently, the relationship between uric acid and AKI in all hospitalized patients was explored. Investigators performed a retrospective database analysis of data for almost 30 years, including 59,219 hospitalized patients, and suggested that SUA levels were an independent risk factor for AKI in men and women [40]. A U-shaped association between them was observed.

It remains unclear whether urate-lowering therapy has beneficial effects in reducing the incidence of AKI. In a cisplatin-induced AKI animal model, lowering SUA with rasburicase (a recombinant urate oxidase enzyme that catalyzes the conversion of uric acid to allantoin) has been shown to decrease tubular injury and the inflammatory response [41]. In a prospective, double-blind, placebo-controlled, randomized trial of 26 hyperuricemic patients undergoing cardiac surgery, there was no observed benefit on postoperative serum creatinine (SCr) in rasburicase versus placebo treatment groups, although novel AKI biomarkers such as urinary NGAL (uNGAL) tended to be lower in rasburicase-treated patients [42].

Several plausible explanations for an increased AKI risk in patients with elevated SUA levels exist [43, 44]. First is a crystal-dependent mechanism, observed primarily in patients who have large tumor burdens and undergo chemotherapy with a rapid increase in serum SUA levels, often to 12 mg/dL or greater. Emerging epidemiological, experimental, and clinical data suggest that uric acid, in concentrations that do not cause intratubular crystal precipitation, may play a role in AKI via crystal-independent mechanisms. Elevated SUA can induce renal vasoconstriction and impair autoregulation, which adversely affects renal blood flow autoregulation and the glomerular filtration rate (GFR). Furthermore, hyperuricemia has a variety of proinflammatory and antiangiogenic effects.

Studies in several settings have demonstrated the prognostic value of SUA levels on AKI development, but more multicenter, prospective studies in broader patient populations are required to confirm the exact relationship between SUA and AKI and the role of potential therapeutic interventions.

### 4.2. Hypoalbuminemia and AKI

Albumin is regarded as a nonspecific marker of nutrition, inflammation, hepatic function, and overall catabolic state [45]. Several factors can influence hypoalbuminemia, including overall nutritional status, specific diseases, and stress responses; thus, controversy persists as to whether it indicates the severity of an underlying disease or is a marker of malnutrition. Albumin has various physiological roles, including binding and transporting diverse toxic endogenous and exogenous compounds, free-radical scavenging, a reservoir for nitric oxide, anti-inflammatory activity, inhibition of apoptosis, and maintenance of capillary membrane permeability, beyond its maintenance of plasma volume by preserving colloid osmotic pressure [46, 47]. All of these suggest renoprotective effects of albumin in AKI, despite a lack of detailed mechanisms. One report found that albumin played an important role as a reactive oxygen species scavenger, protecting the lysophosphatidic acid transporter and preventing apoptosis in renal tubular cells [48]. It had been reported that albumin improved renal blood flow autoregulation through stabilizing endothelial cells [49].

In human clinical evidence, hypoalbuminemia has been reported to be independently associated with an increased risk of AKI in various surgical and other situations [50–55]. A meta-analysis also evaluated the influence of serum albumin levels on the incidence of AKI and mortality in patients with AKI [56]. In total, 11 clinical studies exploring the relationship between albumin and the occurrence of AKI covered a diverse range of medical and surgical cohorts. It was found that each decrement in serum albumin of 10 g/L was associated with a 234% increase in the risk of AKI (pooled OR = 2.34, 95% CI = 1.74–3.14). Another six studies showed that each decrement in serum albumin of 10 g/L was associated with 247% increase in the risk of death after AKI (pooled OR = 2.47, 95% CI = 1.51–4.05). Although these studies used multivariate analyses [50–56], the adjusted confounders were not identical. AKI development and serum concentrations of albumin might be affected by fluid overload, so this should be adjusted for in multivariate analyses. Lee et al. [50, 51] evaluated the volume of fluid infusion during surgery as a covariate in two studies and found that the preoperative albumin level was associated with AKI. Compared to other studies, we found that, regardless of whether the fluid overload was adjusted for, hypoalbuminemia was an independent risk factor for AKI. The biomarkers of inflammation, including C-reactive protein (CRP), eosinophil count, and white blood cell count, indicate the degree of inflammation, which can be adjusted for in multivariate analyses. Lee et al. [50, 51] adjusted for C-reactive protein (CRP) and the result showed that preoperative albumin levels were associated with AKI, the same result as in studies that have not adjusted for CRP [52–55]. Thus, evidence suggests that hypoalbuminemia is a significant independent predictor of both AKI and death following AKI.

Consequently, the correction of hypoalbuminemia would seem to be a reasonable way to prevent AKI and subsequent mortality. A large meta-analysis demonstrated that mortality associated with using albumin-containing solutions could be lower than that with using other fluid regimens [57]. A prospective, single-center, randomized, parallel-arm, double-blind trial concluded that administration of 20% exogenous albumin to patients whose preoperative serum albumin level was < 4.0 g/dL immediately before off-pump coronary artery bypass surgery increased urine output during surgery and reduced the incidence of AKI [58]. There are many possible mechanisms for the beneficial effects of albumin infusion. The correction of hypoalbuminemia expands the intravascular volume, which is likely to confer benefits. The various physiological functions of albumin, especially antioxidant function, may play an important role in its beneficial effects. In addition, the renoprotective effects of albumin itself, such as maintaining renal perfusion and glomerular filtration, mitigating the effect of nephrotoxic medications, cannot be ignored. However, it is difficult to determine which mechanism is the main contributor, because many mechanisms are likely involved in the protective effect of albumin infusion. Future studies need to explore the mechanism of the protective effects of albumin infusion.

The optimal threshold value of albumin below which the risk of AKI becomes absolute remains unknown. The main reason is inconsistency in albumin grouping, which has been based on the characteristics of the study population and the reference values for normal albumin. Albumin has been considered a continuous or categorical variable in different studies. Even when it is considered a categorical variable, the threshold values differ.

However, not all studies have reached the same conclusion. A large, multicenter, randomized study conducted in ICU patients with severe sepsis reported that the correction of hypoalbuminemia by the addition of 20% albumin to crystalloids, as compared with crystalloids alone, had significant benefits on hemodynamics but did not confer a survival benefit [59]. It remains unclear whether albumin administration has any benefit or whether the dose and treatment regimen used is insufficient to have protective effects.

Hypoalbuminemia is a risk factor for AKI and for death after AKI, so serum albumin determinations may be useful to identify patients at increased risk for AKI and for death following AKI. However, whether or not albumin replacement ameliorates that risk lacks convincing evidence and requires confirmation via large clinical investigations in other institutional settings.

### 4.3. Obesity and AKI

Obesity has become an epidemic. There is growing interest in the relationship between obesity and AKI and mortality. Body mass index (BMI) remains the most widely used measure of obesity due to the simplicity of its operational definition and ready accessibility. The prevalence of AKI in obesity has been highlighted in severe AKI requiring dialysis [60], noncardiac surgery [61, 62], cardiac surgery [63, 64], acute respiratory distress syndrome [65], and bariatric surgery [66]. A recent study focused on critically ill patients [67]. In a large database of 5,232 patients with AKI requiring renal replacement therapy (RRT) from 53 Austrian ICUs, the adjusted odds ratios for developing AKI followed a U-shaped pattern, with the lowest morbidity in patients with normal body weight [60]. Another large retrospective analysis of 351,000 patients in the United States published in 2010 showed that, compared to patients of normal weight, obese patients undergoing noncardiac surgery are at a two- to threefold higher risk for AKI complications [61]. A large, single-center study of 154,700 critically ill patients found that the adjusted odds ratios for AKI were 1.18 (95% CI = 1.06–1.31) in overweight, 1.35 (1.19–1.53) in class I obesity (30 ≥ BMI < 35), 1.47 (1.25–1.73) in class II obesity (35 ≥ BMI < 40), and 1.59 (1.31–1.87) in class III obesity (BMI ≥ 40), compared to normal weight [67].

There is a consistent signal regarding the relationship between BMI and the risk of AKI, leading to growing interest in exploring the pathophysiology of obesity-associated AKI. The exact mechanisms may be multifactorial and interactional. Obesity is associated with an increase in proinflammatory cytokines and adipokines and can be regarded as a state of chronic low-grade inflammation. Obesity is also related to an increase in oxidative stress and endothelial dysfunction. It is challenging to assess intravascular volume status and adequacy of fluid resuscitation in the obese patient accurately. Dosing regimens are not aimed primarily at obese populations, but general patients, so there is insufficient knowledge of the efficacy and safety of many drugs that may be nephrotoxic. Many obese patients have other complications, such as hypertension and diabetes, which can increase the risk for AKI, directly or indirectly [68, 69].

Recently, the theory of the so-called “obesity paradox” has emerged; that is, overweight and moderately obese patients seem to have superior survival compared with normal weight and underweight patients. This stemmed from studies in CKD and in hemodialysis and was confirmed by Druml et al. [60]. A multicenter study conducted in AKI patients requiring RRT had the lowest mortality in obese patients (30 ≥ BMI < 35). Soto et al. [65] also showed that increased BMI in patients with ARDS was associated with decreased mortality.

Although unsatisfactory, there is a possible explanation for the “obesity paradox” based on the generation and distribution of uremic toxins in patients. Obese patients have a lower proportion of metabolically active organ mass to total body mass, compared to normal weight patients, which may help to “buffer” the toxins and indirectly confer protective effects [68]. However, a recent cohort study of nearly 15,000 critically ill patients found that each 5 kg/m^2^ BMI increase was not associated with a decreased risk of within-hospital death, which has an OR of 0.98 (95% CI = 0.96–1.09; *p* = 0.59) [67]. Therefore, the relationship between BMI and death in AKI patients remains uncertain. Thus, obesity is an independent risk factor for developing AKI. Studies should explore whether obese patients actually have a survival benefit compared with underweight or normal weight patients.

### 4.4. Anemia and AKI

Anemia and red blood cell (RBC) transfusion have been recognized as potentially modifiable risk factors, often confirmed in surgical patients [70]. A multicenter cohort study of 3,500 adult patients revealed that preoperative anemia and perioperative RBC transfusions were independently and strongly related to AKI after cardiac surgery with cardiopulmonary bypass (CPB) [70]. A systematic review found that preoperative anemia was often related to perioperative allogeneic RBC transfusion [71]; that is, they often occur together in medical practice. In addition, they have been confirmed to have an additive effect on the incidence of postoperative AKI. In a retrospective observational study of 920 patients undergoing CPB [72], per 1 g/dL decrease in hemoglobin concentration a 16% risk of postoperative AKI was added (95% CI = 1.05–1.31, *p* = 0.018). The volume of transfused RBCs was an independent risk factor for AKI. Moreover, the increased risk of AKI associated with transfusion seems to take effect at a hemoglobin level > 8 g/dL (> 5 mmol/L). Karkouti et al. [73] showed that a combination of three risk factors, preoperative and intraoperative anemia and RBC transfusion, was associated with a 2.6-fold (95% CI = 2.0–3.3) increase in the relative risk of AKI after cardiac surgery versus none of these factors.

The mechanisms by which perioperative anemia and RBC transfusions may cause AKI in cardiac surgery have not been determined. The contributory effects of anemia to AKI are likely multifactorial, including reduced renal oxygen delivery, worsening oxidative stress, and impaired hemostasis. The kidneys, especially the renal medulla, are vulnerable to decreased oxygen delivery resulting from anemia. Anemia may be associated with increased renal oxidative stress, due to the important antioxidant functions of RBCs [74]. Anemia impairs hemostatic function via anemia-induced platelet dysfunction; that is, a sufficient (but undetermined) hemoglobin concentration is essential to sustain normal platelet function [75]. Thus, anemia may add to the risk of excessive bleeding, in turn, requiring multiple RBC transfusions, both of which are related to AKI. Transfusions of RBCs can produce adverse effects in susceptible patients, despite the intended therapeutic effect of improving organ function by increasing tissue oxygen delivery. Stored RBCs undergo changes, so transfusions may have negative effects, including impairment of tissue oxygen delivery, promotion of tissue oxidative stress, and activation of leukocytes and the coagulation cascade [76]. All of these can lead to organ dysfunction, with the kidney seeming to be the first to be affected [77].

A low hematocrit during CPB can reliably predict the degree of hemodilution. The lowest acceptable hematocrit during CPB has not been identified definitively. Studies have demonstrated the lowest hematocrit during CPB is a modifiable AKI risk factor. Karkouti et al. [78] showed that the adjusted OR for ARF requiring dialysis support at a hematocrit < 21% was 2.34 times greater than that at a hematocrit of 21–25%. In a recent prospective study, the increased risk of AKI during CPB was associated with a decrease in the lowest hematocrit (RR = 0.933 95% CI = 0.899–0.968, *p* < 0.001), particularly when the lowest hematocrit was ≤ 22% [79]. These studies suggest that the risk of AKI increases dramatically at the lowest hematocrit of < 21-22%. Hemodilution obviously decreases the oxygen-carrying capacity of the blood. However, the lower blood viscosity can improve the regional blood flow, minimizing the need for blood perfusion. The underlying pathogenesis of AKI is considered to involve systemic inflammation, arising from ischemia, or impaired oxygen delivery to the hypoxic kidney medulla [80].

### 4.5. Hyperglycemia and AKI

In recent years, more research has focused on the important role of hyperglycemia in AKI. Among critically ill patients who received total parenteral nutrition (TPN) in ICUs, the OR (1.47, 95% CI = 1.00–2.17, *p* = 0.05) of ARF increased with higher glucose levels [81]. Among patients undergoing cardiac surgery, preoperative capillary glucose above 140 mg/dL could accurately predict AKI following cardiac surgery [82]. In patients with acute MI (AMI), elevated admission plasma glucose was associated with a greater risk for AKI (OR = 1.10, 95% CI = 1.03–1.18, *p* = 0.02) after adjusting for confounding factors [83]. Among patients with AMI undergoing coronary angiography, preprocedural glucose was an independent predictor of CI-AKI in patients without known diabetes, but not in patients with diabetes [84]. Another study targeting patients who underwent transcatheter aortic valve implantation (TAVI) concluded that those with postprocedural hyperglycemia had a twofold higher occurrence of AKI, regardless of the presence of DM and CKD [85].

Recent studies have also revealed emerging associations between variation in glucose levels and AKI. A recent prospective cohort study showed that the magnitude of perioperative glycemic variability was an independent predictor of AKI in patients undergoing elective coronary artery bypass grafting (CABG) [86]. Another study conducted in liver transplantation recipients showed perioperative glucose fluctuations, but not hyperglycemia per se, and the risk of postoperative AKI were positively correlated [87]. This highlights the need to avoid wide perioperative glycemic fluctuations.

Researches have shown that hyperglycemia should be treated; however, the optimal target levels of glucose at which the risk of AKI becomes absolute and should always be treated remain uncertain. It is also not clear whether the optimal target levels of glucose should be the same in different degrees of illness or different indications for ICU admission [88]. Kavanagh et al. [88] suggested that plasma glucose levels above 180 mg/dL should be treated. However, the level of hyperglycemia that should be treated has not been clearly established. Increasing numbers of studies have found that tight glycemic control (80–110 mg/dL) does not confer survival benefits and may increase the risk of hypoglycemia and mortality [89]. Therefore, the KDIGO AKI guidelines suggest the target plasma glucose concentration should be between 110 and 140 mg/dL in critically ill patients (6.1–8.3 mmol/l). Considering the potential adverse effects and limited studies of oral antidiabetic agents, insulin is generally recommended to AKI and critically ill patients.

Suggested mechanisms for a causal relationship between hyperglycemia and AKI included the following [85, 90]. Hyperglycemia is accompanied by stress responses, mediated by the release of cortisol and catecholamines. Hyperglycemia increases oxidative stress and suppresses flow-mediated vasodilatation, resulting in medullary hypoxia and ischemia. Moreover, acute hyperglycemia may lead to volume depletion and then increase the risk for prerenal azotemia due to osmotic diuresis. However, acute glucose fluctuations are increasingly regarded as a more specific trigger of oxidative stress than chronic sustained hyperglycemic states [91]; as such, glycemic fluctuation could aggravate unfavorable effects. It is certain that hyperglycemia is associated with the development of AKI, but the real role of hyperglycemia in AKI remains unclear.

## 5. Conclusions

In conclusion, AKI is a worldwide public health problem due to its high morbidity, poor prognosis, and lack of a cure. Understanding the risk profile of AKI may be of benefit in the early prevention and treatment of AKI. Both the patient's susceptibility and the type and extent of exposure determine the risk of AKI. Many risk factors are known but are mostly not readily modifiable. Recent studies have shown that hyperuricemia, hypoalbuminemia, obesity, anemia, and hyperglycemia may be novel predictors of AKI. However, as to which of these are useful targets for interventions requires further exploration.
